# Coaxial-Robotic Single-Site Myomectomy: Surgical Outcomes Compared with Robotic Single-Site Myomectomy by Propensity Score Matching Analysis

**DOI:** 10.3390/jpm13010017

**Published:** 2022-12-22

**Authors:** Su Hyeon Choi, Seyeon Won, Nara Lee, So Hyun Shim, Mi Kyoung Kim, Mi-La Kim, Yong Wook Jung, Bo Seong Yun, Seok Ju Seong

**Affiliations:** 1 Department of Obstetrics and Gynecology, CHA Gangnam Medical Center, CHA University School of Medicine, Seoul 06135, Republic of Korea; 2 Department of Obstetrics and Gynecology, CHA Ilsan Medical Center, CHA University School of Medicine, Goyang 06135, Republic of Korea

**Keywords:** uterine myomectomy, robotic surgical procedures, laparoscopy, uterine fibroids

## Abstract

Background: The aim of this study was to introduce a coaxial-robotic single-site myomectomy (C-RSSM) technique to compensate for the shortcomings of robotic single-site myomectomy (RSSM) using semi-rigid instruments and to compare the surgical outcomes of C-RSSM and RSSM. Methods: The medical records of 13 consecutive women who had undergone C-RSSM and 131 consecutive women who had undergone RSSM were retrospectively reviewed. Patient characteristics and surgical outcomes after propensity score matching were evaluated and compared between the two groups. Results: According to the propensity score matching results, the C-RSSM group had a lower estimated blood loss (75.0 vs. 210.5 mL, *p* = 0.001) and a shorter operating time (101.0 vs. 146.1 min, *p* = 0.008) relative to the RSSM group. In RSSM, there was one case of conversion to conventional laparoscopy and four cases of conversion to the multi-site robotic approach. There was no case of conversion from C-RSSM to conventional laparoscopy or the multi-site robotic approach. Conclusions: C-RSSM was found to be associated with shorter operative time and lower estimated blood loss. However, further prospective studies are needed to confirm these advantages.

## 1. Introduction

Uterine leiomyomas, commonly known as fibroids, are among the most common benign pelvic tumors, being diagnosed in up to 70–80% of women during their lifetime. Affecting a significant proportion of reproductive-aged women, they may cause menorrhagia, pelvic pain or pressure symptoms, and can even adversely affect reproductive outcomes [1]. Myomas are the first indication for hysterectomy, but myomectomy is the most suitable surgical option for women who want to preserve their fertility potential [2]. The surgical options available for myomectomy have improved, evolving from open-abdominal myomectomy to minimally invasive surgery such as laparoscopic myomectomy or robot-assisted laparoscopic myomectomy (RALM) [3,4]. Robotic technology has shown feasibility in both gynecological and reproductive surgery applications [5]. Recent studies have shown that RALM has advantages over laparoscopic myomectomy in terms of blood loss, peri- or postoperative transfusion, and length of hospital stay [6,7]. Additionally, patients’ desire to reduce surgical scarring and improve their overall cosmetic satisfaction has stimulated interest in laparo-endoscopic single-site (LESS) surgery [8]. While many studies have established the feasibility of LESS surgery, it remains challenging from the technical standpoint, due to systemic limitations that include inter-instrument fighting, camera–platform instability, limited straight-instrument range of motion, and lack of instrumentation triangulation [9]. A promising alternative, namely robotic single-site myomectomy (RSSM), offers a means of overcoming the disadvantages of single-site myomectomy, and indeed, its feasibility and safety have been proven in many studies [10,11,12,13]. However, its limited range of motion, with weak traction power for semi-rigid single-site robotic instruments and the still-existing problem of inter-instrument fighting, remain obstacles [14].

Coaxial-RSSM (C-RSSM) was designed as an alternative single-site surgical modality that can compensate for the limitations of RSSM. Specifically, its coaxial system can provide stronger traction power suitable for 8 mm conventional robotic instrumentation, as well as improved suturing strength relative to RSSM. Of course, in the case of C-RSSM, there are inevitably some restrictions in terms of the movement of the instruments [15]. Given the lack of any relevant investigations in the literature, the present study was designed and undertaken to confirm the safety and feasibility of C-RSSM by the comparative analysis of clinical characteristics and surgical outcomes between it and RSSM.

## 2. Materials and Methods

All women diagnosed with symptomatic uterine fibroids who had undergone, at a single institution, C-RSSM (n = 13) between October 2021 and September 2022 (by a single surgeon) or RSSM (n =131) between August 2015 and February 2019 (by five surgeons) were included in this retrospective cohort study. Patient selection criteria was generally that the number of myomas was less than three, and the size of the largest myoma was less than 10 cm. The myoma was screened for possible malignancy, and an MRI was performed when necrotic features were seen on ultrasound findings. Institutional Review Board approval was obtained (GCI 2022-09-008). The surgical procedures were performed using the da Vinci Xi Surgical System (Intuitive Surgical, Sunnyvale, CA, USA). All six surgeons had extensive experience in robotic surgery (>200 operations).

### 2.1. Data Characteristics

Patient data including age, body mass index (BMI), parity, previous abdominal surgical history including laparoscopy and/or laparotomy, and myoma features were extracted from the medical records. We checked the preoperative hemoglobin when the operation was scheduled (within 1 month of the operation) and the postoperative hemoglobin at the first postoperative day. The total operation time was defined as the interval between the start of skin incision and the completion of skin closure.

### 2.2. Surgical Procedures

All of the patients were administrated general anesthesia in the lithotomy position. A Foley catheter and a RUMI uterine manipulator (CooperSurgical Inc., Trumbull, CT, USA) were both inserted preoperatively. During surgery, frozen biopsies were performed when the leiomyosarcoma was suspicious, showing features such as necrotic change. It was not present in the C-RSSM group, but three cases were performed in the RSSM group, which were all benign.

### 2.3. Coaxial-Robotic Single Site Myomectomy (C-RSSM)

**Figure 1:**
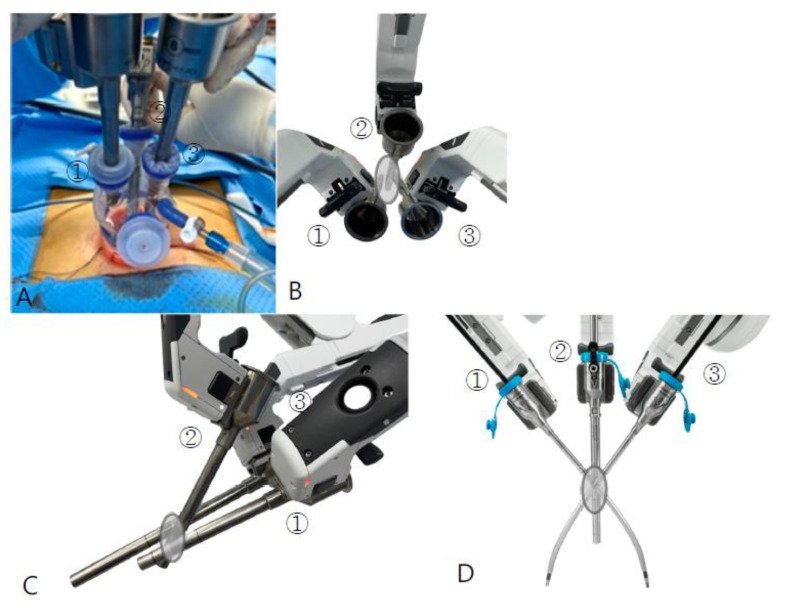
Placement of cannulas for C-RSSM (**A**–**C**) and RSSM (**D**); the gray circle represents a single incision port. ①, ② and ③ are connected to arm 1, arm 2, and arm 3. (**A**,**B**,**D**: View from the patient’s head, **C**: View from beside the patient); C-RSSM, Coaxial-robotic single-site myomectomy; RSSM, robotic single-site myomectomy.

**Figure 2:**
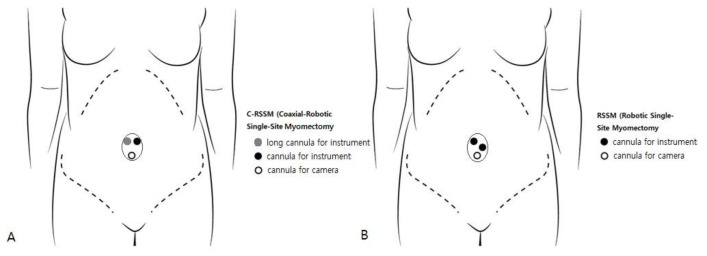
Placement of cannulas for C-RSSM (**A**) and RSSM (**B**) (view from above); C-RSSM, coaxial-robotic single-site myomectomy; RSSM, robotic single-site myomectomy.

**Figure 3:**
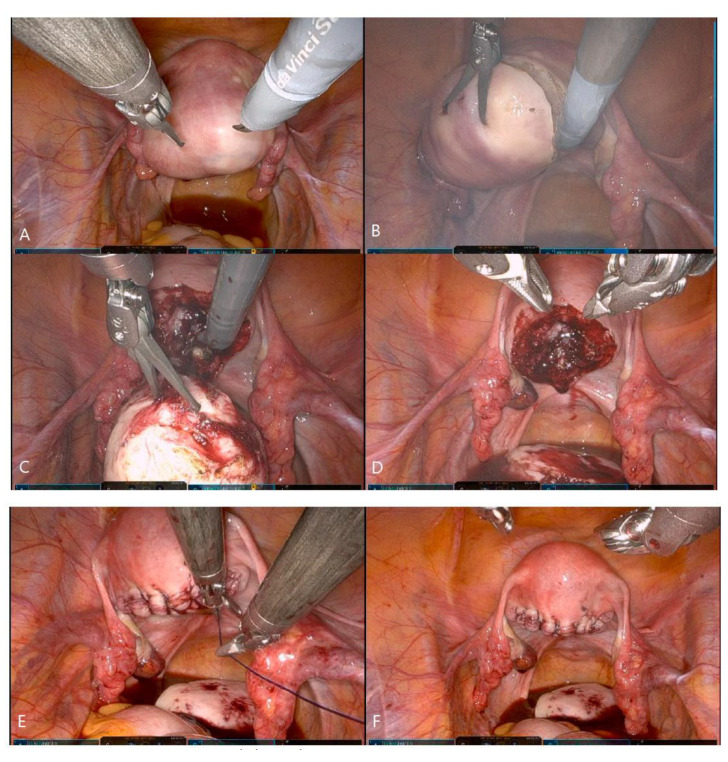
Myomectomy in C-RSSM; C-RSSM, coaxial-robotic single-site myomectomy. (**A**) Robotic camera view (the camera is illuminated from rear to front, from bottom to top, and the instruments are positioned relatively up front). (**B**) Within a limited range, traction and countertraction of myoma. (**C**) Myoma traction with a tenaculum grasper. (**D**) After myoma enucleation. (**E**) Suturing the uterus. (**F**) After suturing.

First, the single-site port of entry was established using a wound retractor with a 2.3-to-2.5 cm incision at the umbilicus, which is the same procedure as in RSSM. Then, the 4-channel glove port (Nelis, Seoul, Korea) was inserted through the umbilical incision. The three 8 mm robotic cannulas, a regular-length 10 cm cannula for the camera port, a 10 cm cannula for one instrument, and a long 15 cm cannula for the other instrument were placed in each channel of the glove port (Figure 1A–C). The patient-side cart was docked onto the three cannulas, arm 1—the 10 cm cannula, arm 2—the camera-port cannula, and arm 3—the long cannula. To minimize external fighting of the robotic arms and instruments, it is helpful to operate with robotic arms 1 and 3 while arm 2 is folded to the back of the cart. While maintaining the same incision size as with RSSM but using thicker and more rigid cannulas placed into the incision, we designed two important extra “steps” in order to successfully complete the C-RSSM setup and ensure docking success. The first step is the use of a camera scope for a 30° upside view. So, the camera cannula stands toward the caudal side and is relatively steeper relative to the ground than the two instrument cannulas. The two instrument cannulas and the camera cannula form an inverted triangle (Figure 2A). However, the cannula is gathered in a single-incision, and so when the arms are docked, fighting continues to be a problem during the procedure. For this reason, we designed our second important extra step, particularly because the range of motion of the instrument is very limited: the use of a long 15 cm cannula allows for one instrument arm to sit higher above the abdominal wall relative to the other instrument arm and the camera arm, further decreasing the chance of fighting. In summary, (1) the camera is designed to stand steeply toward caudal side so that the field of view is at an angle from bottom to top, in order to secure space by laying down both instrument arms and avoid, thereby, fighting; and, (2) in order to increase the range of movement of the instrument tips, a long cannula is used to maintain distance between the two arms. Robotic arm 1 was loaded with a robotic tenaculum or fenestrated bipolar forceps, while robotic arm 3 was loaded with wristed monopolar curved scissors or mega-needle driver. After inspecting the abdominal cavity, a vasopressin solution of 0.25-U/mL concentration was injected into the target uterine tissue. Figure 3 shows the process of retracting the myoma while pulling powerfully with the tenaculum as well as the process of vigorously suturing it with the mega-needle driver using barbed sutures. The myoma was retrieved through the umbilical incision by manual scalpel morcellation in a surgical bag. The myoma was retrieved through the umbilical incision by scalpel morcellation. The peritoneum, fascia, and subcutaneous layers were closed using 1-0 vicryl (Ethicon), and the skin was closed using 3-0 vicryl sutures.

### 2.4. Robotic Single-Site Myomectomy (RSSM)

First, the single-site port of entry was established using a wound retractor with a 2.3-to-2.5 cm incision at the umbilicus. Then, the da Vinci single-site silicon port was inserted through the umbilical incision, through which port the 8.5 mm robotic camera cannula, two curved 5 mm long robotic cannulas (250–300 mm in length) and a 5 or 10 mm assist cannula were introduced. Next, the robotic system was docked vertically, and a 30° camera scope for the downside or upside view was introduced. After docking, semi-rigid robotic instruments, specifically a monopolar hook and a fenestrated bipolar forceps, were placed in the robotic arms (Figure 1D and Figure 2B). After inspecting the abdominal cavity, a diluted solution of vasopressin was injected. An incision into the uterus was made using the monopolar hook, and the myoma was enucleated. After enucleation, the monopolar hook was changed to a wristed needle holder. The uterine wall was sutured layer to layer with continuous barbed sutures. Finally, the teleoperator was undocked, and the myoma was retrieved through the umbilical incision manual scalpel morcellation in a surgical bag. The peritoneum, fascia, and subcutaneous layers were closed using 1-0 vicryl, and the skin was closed using 3-0 vicryl sutures. Myoma retrieval and abdominal wall suturing were performed in the same way as in C-RSSM.

### 2.5. Statistical Analysis

All continuous data were expressed as the mean ± standard deviation (SD), and categorical data were reported as absolute numbers or percentages. Frequency distributions were compared using the χ^2^ test, and the mean or median values were compared using Student’s t- or Mann–Whitney U-test as appropriate after ascertaining the normality of distribution via the Kolmogorov–Smirnov or Shapiro–Wilk test. All of the calculated *p* values were two-sided, and *p* < 0.05 was considered statistically significant. The data were analyzed using SPSS version 24.0 (IBM Inc., Armonk, NY, USA). 1:n propensity score matching (PSM) with a nearest-neighbor matching algorithm was performed to minimize selection bias. The number of myomas, the size of the largest myoma, tumor weight, and BMI were selected as the PSM variables, because those were the variables that were statistically different between the groups or could affect postoperative outcomes. Based on the propensity scores, 10 patients who had undergone C-RSSM were matched with 19 patients who had undergone RSSM.

## 3. Results

### 3.1. Baseline Characteristics

The patients’ baseline characteristics are provided in Table 1. In the RSSM group relative to C-RSSM, a smaller size of largest myoma (6.2 ± 1.7 vs. 7.2 ± 1.3 cm, *p* = 0.011) and a higher BMI (21.7 ± 3.3 vs. 19.6 ± 1.83, *p* = 0.011) were found. The highest BMI was 23.2 in C-RSSM group and 34.7 in RSSM group, and there were no problems for trocar placement. Otherwise, there were no significant inter-group differences in total myoma number (2.7 ± 2.2 vs. 1.8 ± 1.4, *p* = 0.195), tumor weight (114.9 ± 81.3 vs. 155.8 ± 89.5, *p* = 0.073), or type and location of myoma. Table 2 shows the post-PSM baseline characteristics. After PSM, there was no difference in BMI, which earlier had shown a significant difference between the two groups. In fact, as is apparent from the table, there were no significant inter-group differences of any kind.

### 3.2. Surgical Outcomes

Table 3 summarizes the surgical outcomes. In the total data, C-RSSM showed a shorter operation time (100.8 ± 18.8 vs. 141.4 ± 54.4 min, *p*= 0.011) and a lower estimated blood loss (EBL) (84.6 ± 47.3 vs. 212.3 ± 189.8 mL, *p* <0.001) relative to RSSM. After matching for the number of myomas, the size of the largest myoma, tumor weight, and BMI, C-RSSM was found to be associated with significantly shorter total operation time relative to RSSM (101.0 ± 20.5 vs. 146.1 ± 47.5 min, *p* = 0.008). Similarly, C-RSSM was associated with significantly lower EBL relative to RSSM (75.0 ± 35.4 vs 210.5 ± 129.7 mL, *p* = 0.001). The hemoglobin decrement (1.8 ± 0.7 vs 2.0 ± 1.2 g/dL, *p* = 0.924) and duration of hospital stay (4.3 ± 0.6 vs. 4.3 ± 0.5 days, *p* = 0.386) did not differ between the groups. One patient in the C-RSSM group and one in the RSSM group experienced postoperative paralytic ileus. With conservative management, bowel function was restored. There was no intraoperative complication or conversion to laparotomy in either group. There was one case of conversion from RSSM to conventional laparoscopy owing to severe pelvic adhesions, and four cases of conversion from RSSM to the multi-site robotic approach due to technical difficulties. Among them, one case was due to an omental adhesion, and the others were due to the difficult location of the myoma (posterior and deep, less than FIGO type 4). There was no case of conversion from C-RSSM to conventional laparoscopy or the multi-site robotic approach. After PSM, one case (5.3%) was converted from RSSM to a multi-site robotic approach, and no cases (0%) were converted from C-RSSM to another surgical method. 

## 4. Discussion

LESS surgery has been performed for decades for both minor and major gynecological procedures [16,17,18]. Due to the reconstructive and suture-intensive nature of myomectomy, it has been difficult to apply the single-site approach [19,20]. In fact, laparoscopic single-site myomectomy (LSSM), lacking triangulation, has challenging ergonomics; it demands advanced surgical skills to overcome inter-instrument fighting [21,22]. The idea of combining LSSM with robotic surgical systems (e.g., RSSM) seems a promising alternative for overcoming such technical difficulties [23,24]. However, the disadvantages of RSSM are as follows. First, its semi-rigid robotic instruments lack sufficient mechanical power for traction and countertraction during the enucleation of myomas or for the reconstruction of the uterus by layer-to-layer suturing. Second, to mechanically support RSSM’s semi-rigid instruments, relatively long cannulas are needed. Considering the length of the instrument tip and the long cannula located below the umbilicus level, the enucleation of large myomas is indeed difficult due to the limited workspace.

To overcome the limitations of RSSM, we designed a new surgical technique, namely “hybrid RSSM (H-RSSM)”. H-RSSM demonstrated a shorter operation time and lower EBL relative to RSSM [25]. However, H-RSSM still had certain disadvantages, such as requiring significant transition time from laparoscopy to the robot mode, the continuation of bleeding during that time, and a greater amount of bleeding for a larger number of myomas removed. In light of these stubborn difficulties, we carefully devised our new coaxial-RSSM (C-RSSM) approach as a new surgical technique. With C-RSSM, since there is no need for the laparoscopy-to-robot transition, there is no delay time before suturing, and in addition, there is sufficient traction and countertraction for the enucleation of myomas and suture-intensive surgery.

As briefly described above, there are two key requirements for uterine myomectomy: first, traction power for removal of myomas, and second, suturing strength. In RSSM, for the enucleation of myomas, a monopolar hook and a fenestrated bipolar forceps are used, which are semi-rigid and, relative to the tenaculum, lack power due to poor fixation. With C-RSSM, using an 8 mm tenaculum, it is possible to apply sufficient traction strength. After the removal of myomas from the uterus, the uterus should be firmly sutured layer-to-layer. With RSSM, the suturing strength is weak due to the use of the 5 mm semi-rigid instrument; however, in C-RSSM, the suturing strength is strong, thanks to the 8 mm conventional rigid instrument. Given RSSM’s poor traction and low suturing strength, surgery takes a considerable time, incurs significant EBL, and the overall surgical difficulty is increased relative to C-RSSM [14,25]. In the present study, despite the largest myoma size being significantly larger than in RSSM, C-RSSM showed a shorter operation time and lower EBL. Even after PSM, C-RSSM was found to be associated with a significantly shorter total operation time and lower EBL relative to RSSM. These advantages are thought to be the results of the sufficient traction and suture-intensive surgery provided by C-RSSM.

C-RSSM holds great promise for resolving the ergonomic challenges imposed by the restrictive range of motion, in-line work, and restricted vision of conventional RSSM [26,27]. This notwithstanding, C-RSSM also has disadvantages. Design efforts had been made to reduce fighting as much as possible by using two cannulas of different lengths and a 30° upside view; however, since thick and straight cannulas enter into a small single hole, movements are somewhat restricted, and moreover, accessing myomas located on the anterior-lower wall incurs some difficulty. However, in this study, there was no difference in myoma location between the two groups, and good surgical outcomes were shown in C-RSSM group. However, provided that C-RSSM’s shortcomings are recognized and that indications for its application are well defined, it will be more widely utilized for its many benefits.

To the best of our knowledge, this is the first comparative study to have evaluated surgical outcomes of C-RSSM versus those of RSSM. The most relevant study conducted thus far reported on the experience of the first 21 surgeries using C-RSSM [15]. In that study, the umbilicus incision length was 3–4 cm. In the present study, by contrast, C-RSSM procedures were successfully performed with a smaller incision, i.e., the same incision length as in RSSM. Thereby, the cosmetic results are better than previously, and are comparable those of RSSM.

This study has several limitations. First, this was a retrospective study with a small sample size, especially for the C-RSSM group. Second, the operative time was not evaluated in detail. If the myoma enucleation time and suturing time were measured and compared, more meaningful results could have been drawn. Third, the surgical method selection was not randomized. Therefore, selection bias may have affected the results.

In conclusion, this study is the first to have assessed surgical outcomes of C-RSSM versus those of RSSM. C-RSSM, having a single incision site, was associated with a shorter operative time and lower EBL. The possible as-yet-unrevealed advantages of C-RSSM should be investigated in further prospective studies with large sample sizes.

## Figures and Tables

**Table 1:** Baseline characteristics of myomectomy patients.

Characteristics	C-RSSM (n = 13)	RSSM (n = 131)	*p*
Age, years	35.1 ± 5.9	36.9 ± 6.1	0.211
BMI, kg/m^2^	19.6 ± 1.83	21.7 ± 3.3	0.011
Parity	0.15 ± 0.38	0.45 ± 0.82	0.267
Previous abdominal surgery			0.704
No	10 (76.9)	108 (82.4)	
Yes	3 (23.1)	23 (17.6)	
Peritoneal adhesion			>0.999
No	12 (92.3)	119 (90.8)	
Yes	1 (7.7)	12 (9.2)	
Concurrent surgery			0.833
No	11 (84.6)	110 (84.0)	
Ovarian cystectomy	1 (7.7)	14 (10.7)	
USO	0 (0)	1 (0.8)	
Focal adenomyomectomy	1 (7.7)	6 (4.6)	
Total myoma, n	1.8 ± 1.4	2.7 ± 2.2	0.195
Largest myoma Size, cm	7.2 ± 1.3	6.2 ± 1.7	0.011
Location			0.898
Anterior	7 (53.8)	57 (43.5)	
Posterior	5 (38.5)	53 (40.5)	
Fundal	1 (7.7)	17 (13.0)	
Anterior fundal	0 (0)	2 (1.5)	
Posterior fundal	0 (0)	2 (1.5)	
Type (FIGO classification)			0.057
Submucosal (1–2)	1 (7.7)	4 (3.1)	
Intramural (3–4)	4 (30.8)	67 (51.1)	
Imtramural-subserosal (5)	5 (38.5)	31 (23.7)	
Subserosal (6)	1 (7.7)	22 (16.8)	
Pedunculated (7)	2 (15.4)	2 (1.5)	
Intraligamentary (8)	0 (0)	5 (3.8)	
Tumor weight, g	155.8 ± 89.5	114.9 ± 81.3	0.073

Note: Values are presented as number (%), median (range), or mean ± standard deviations. Abbreviations: BMI, body mass index; USO, unilateral salpingo-oophorectomy; FIGO, International Federation of Gynecology and Obstetrics; C-RSSM, coaxial-robotic single-site myomectomy; RSSM, robotic single-site myomectomy.

**Table 2:** Baseline characteristics of myomectomy patients after propensity score matching (PSM).

Characteristics	C-RSSM (n = 10)	RSSM (n = 19)	*p*
Age, years	35.7 ± 6.2	35.8 ± 6.2	0.713
BMI, kg/m^2^	20.2 ± 1.7	20.2 ± 2.1	0.947
Parity	0.20 ± 0.42	0.32 ± 0.58	0.666
Previous abdominal surgery			>0.999
No	8 (80.0)	16 (84.2)	
Yes	2 (20.0)	3 (15.8)	
Peritoneal adhesion			>0.999
No	9 (90.0)	18 (94.7)	
Yes	1 (10.0)	1 (5.3)	
Concurrent surgery			0.551
No	8 (80.0)	15 (78.9)	
Ovarian cystectomy	1 (10.0)	4 (21.1)	
USO	0 (0)	0 (0)	
Focal adenomyomectomy	1 (10.0)	0 (0)	
Total myoma, n	1.9 ± 1.5	2.0 ± 1.3	0.980
Largest myoma Size, cm	7.0 ± 1.2	6.9 ± 1.2	0.946
Location			0.493
Anterior	5 (50.0)	12 (63.2)	
Posterior	4 (40.0)	7 (26.1)	
Fundal	1 (10.0)	0 (0.0)	
Anterior fundal	0 (0)	0 (0)	
Posterior fundal	0 (0)	0 (0)	
Type (FIGO classification)			0.254
Submucosal (1–2)	1 (10.0)	1 (5.3)	
Intramural (3–4)	2 (20.0)	8 (42.1)	
Imtramural-subserosal (5)	5 (50.0)	4 (21.1)	
Subserosal (6)	0 (0)	3 (15.8)	
Pedunculated (7)	2 (20.0)	1 (5.3)	
Intraligamentary (8)	0 (0)	2 (10.5)	
Tumor weight, g	140.5 ± 89.1	137.1 ± 86.4	0.783

Note: Values are presented as number (%), median (range), or mean ± standard deviations. Abbreviations: BMI, body mass index; USO, unilateral salpingo-oophorectomy; FIGO, International Federation of Gynecology and Obstetrics; C-RSSM, coaxial-robotic single-site myomectomy; RSSM, robotic single-site myomectomy.

**Table 3:** Surgical outcomes and morbidity before and after propensity score matching (PSM).

Characteristics	Total Data			In PSM Data		
	C-RSSM (n = 13)	RSSM (n = 131)	*p*	C-RSSM (n = 10)	RSSM (n = 19)	*p*
Operative time, min	100.8 ± 18.8	141.4 ± 54.4	0.011	101.0 ± 20.5	146.1 ± 47.5	0.008
EBL, mL	84.6 ± 47.3	212.3 ± 189.8	<0.001	75.0 ± 35.4	210.5 ± 129.7	0.001
Hemoglobin decrement, g/dL	2.0 ± 1.1	1.4 ± 1.2	0.23	2.0 ± 1.2	1.8 ± 0.7	0.924
Transfusion			>0.999			>0.999
No	13 (100)	127 (96.9)		10 (100)	19 (100)	
Yes	0 (0)	4 (3.1)		0 (0)	0 (0)	
Hospital stay, days	4.7 ± 1.4	4.7 ± 0.8	0.262	4.3 ± 0.5	4.3 ± 0.6	0.386
Conversion			>0.999			>0.999
No	13 (100)	126 (96.2)		10 (100)	18 (94.7)	
Laparotomy	0 (0)	0 (0)		0 (0)	0 (0)	
Laparoscopy	0 (0)	1 (0.8)		0 (0)	0 (0)	
Multi-site	0 (0)	4 (3.1)		0 (0)	1 (5.3)	
Complications			0.173			>0.999
None	12 (92.3)	130 (99.2)		10 (100)	19 (100)	
Ileus	1 (7.7)	1 (0.8)		0 (0)	0 (0)	
Fever > 3 days	0 (0)	0 (0)		0 (0)	0 (0)	
Wound dehiscence	0 (0)	0 (0)		0 (0)	0 (0)	

Note: Values are presented as number (%), median (range), or mean ± standard deviations. Abbreviations: EBL, estimated blood loss; C-RSSM, coaxial-robotic single-site myomectomy; RSSM, robotic single-site myomectomy.
